# New techniques for studying neurodevelopment

**DOI:** 10.12703/r/9-17

**Published:** 2020-11-25

**Authors:** Augusto Escalante, Rocío González-Martínez, Eloísa Herrera

**Affiliations:** 1 Instituto de Neurociencias (Consejo Superior de Investigaciones Cientíﬁcas-Universidad Miguel Hernández, CSIC-UMH), Campus San Juan, Av. Ramón y Cajal s/n, Alicante 03550, Spain

**Keywords:** Neural development tools, Light sheet microscopy, Clearing, scRNAseq, Machine learning

## Abstract

The extraordinary diversity, variability, and complexity of cell types in the vertebrate brain is overwhelming and far exceeds that of any other organ. This complexity is the result of multiple cell divisions and intricate gene regulation and cell movements that take place during embryonic development. Understanding the cellular and molecular mechanisms underlying these complicated developmental processes requires the ability to obtain a complete registry of interconnected events often taking place far apart from each other. To assist with this challenging task, developmental neuroscientists take advantage of a broad set of methods and technologies, often adopted from other fields of research. Here, we review some of the methods developed in recent years whose use has rapidly spread for application in the field of developmental neuroscience. We also provide several considerations regarding the promise that these techniques hold for the near future and share some ideas on how existing methods from other research fields could help with the analysis of how neural circuits emerge.

## Moving forward

Long gone are the times when budding neuroscientists would picture themselves working like Don Santiago Ramón y Cajal using only a simple microscope and a shelf full of chemical reagents^1^. Today, neuroscientists of all stripes, including those working on the development of the nervous system, are taking advantage of the breadth of new methods and technologies that Don Santiago could have only dreamt about. These methods accelerate our capacity to collect and analyse biological information in large and complex specimens. For instance, we can now reconstruct in three dimensions (3D) the complete peripheral nervous system of a cleared lizard embryo^2^ or obtain a transcriptomic map of gene expression at the single cell (or nucleus) resolution from almost any tissue and species, including humans. Science and technology have been interconnected always, and advances in one historically translate into important progress in the other. Today, the number of available advanced techniques can be overwhelming. In this review, we discuss some recently developed techniques that are currently becoming common in laboratories studying neural development.

## Shining light through the 3D embryonic nervous system

Our capacity to document the 3D organisation of the embryonic brain to understand the basic mechanisms underlying circuit formation has been limited until very recently. Studies on the development of the nervous system of most vertebrates have traditionally relied on histological sectioning methods or open book preparations that enable the visualisation of two-dimensional organisation of the axon tracts in the samples under epifluorescence or confocal microscopes. These approaches, which are based on the observation of selected slices or planes of observation (a process that can inherently introduce biases), provide only partial information about the sample. Even though 3D imaging of small embryos had been performed for many years using wide-field and confocal microscopy^3^, these techniques were very slow and do not scale well to larger embryos or postnatal tissues. This situation began to change with the appearance of light sheet fluorescence microscopy (LSFM). The main advantage of LSFM is the high speed of acquisition and the ability to image large sample sizes that were unpractical to image with conventional microscopes. LSFM was initially used in the field of colloidal chemistry^4^, and about 30 years ago it was adapted to biology to visualise guinea-pig cochleas in 3D^5^. LSFM combines the speed of wide-field imaging with optical sectioning and low photobleaching. In conventional fluorescence microscopy, the entire thickness of the sample is illuminated in the same direction as the detection optics, and, as such, the regions outside the detection focal plane of the objective are potentially damaged by extraneous out-of-focus light that increases the photobleaching. In contrast, in LSFM, the sample is illuminated from the side, perpendicular to the direction of observation, thereby placing the excitation light only where it is required. Therefore, this technique enables the visualisation of tissue samples by shining a sheet of light through the specimen, generating a series of images that can then be digitally reconstructed thanks to the development of sophisticated algorithms and huge improvement in the capacity of computers to store and analyse data^6^. In developmental biology, LSFM was used for the first time to visualise the transparent tissues of zebrafish and *Drosophila* embryos in 3D *in vivo*. About 8 years ago, Tomer and colleagues were able to visualise the development of the *Drosophila* ventral nerve cord for the first time^7^, and Ahrens and co-authors measured the activity of single neurons in the brain of larval zebrafish embryos^8^ using *in vivo* light sheet microscopy.

However, what eventually enabled LSFM to be used for the analysis of the nervous system was the remarkable improvement in brain clearing techniques. The rapid optimisation of clearing protocols has expanded the application of LSFM in the field of developmental neuroscience in the last 4 to 5 years. Since then, a myriad of different approaches to perform tissue clearing have been developed; these approaches vary based on the type of chemical reagents used and depend on the size of the samples. Although exhaustive reviews about the diversity of clearing protocols have been published^9–11^, it is worth mentioning the variants of the CUBIC and DISCO series because their excellent results and easy performance ultimately exalted them as the most widespread methods for brain clearing (see https://idisco.info and http://cubic.riken.jp).

**Figure 1.**
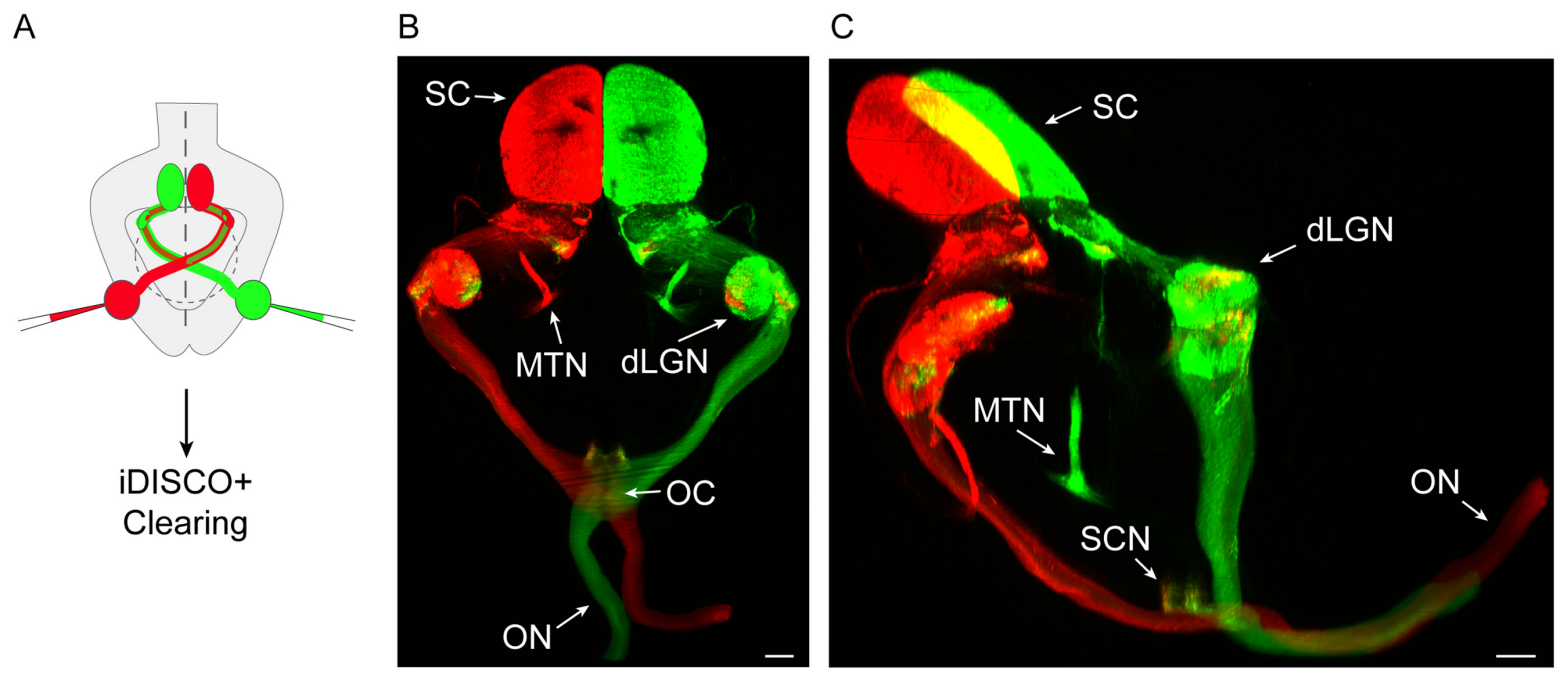
Three-dimensional (3D) view of retinal axons projecting to the visual nuclei within the mouse brain. **A.** Scheme of the experimental approach. A postnatal mouse is injected with fluorescent tracers of different colours in each eye and then processed through the iDISCO+ clearing protocol^24^. **B.** Dorsal view of a light sheet fluorescence microscope (LSFM)-acquired 3D image stack from the whole brain of a mouse injected with different colour tracers into each eye. dLGN, dorsal lateral geniculate nucleus; OC, optic chiasm; ON, optic nerve; MTN, medial terminal nucleus; SC, superior colliculus. Scale bar: 300 µm. **C.** Mediolateral view of an LSFM-acquired 3D image stack from the whole brain of a mouse injected with different colour tracers into each eye. dLGN, dorsal lateral geniculate nucleus; MTN, medial terminal nucleus; ON, optic nerve; SC, superior colliculus; SCN, suprachiasmatic nucleus. Scale bar: 300 µm.

Now that we have methods to make the mammalian brain transparent and visualise it in 3D, a new world has opened up. The combination of tissue clearing and LSFM in neurodevelopmental research is rapidly contributing to important findings in the areas of cell migration and axon pathfinding. One representative example of such advances is the discovery of a small population of neurons in human embryos that secrete gonadotropin-releasing hormones and follow two different pathways of migration beyond the hypothalamus^12^. In axon guidance studies, this combination approach is proving to be extremely useful for visualising neuronal axons growing across the whole embryo and for detecting pathfinding defects in mutants of different members of the main families of axon guidance molecules^13^. It has been possible to visualise for the first time the development of the peripheral nervous system and the innervation patterns of human embryos^14^. Now, the power of combining these approaches with axonal tracings^15^ or antibody staining after functional manipulations (transgenesis^16^, *in utero* electroporation, or viral transduction^17^) holds the promise of interesting times ahead (Figure 1). The possibility of applying these techniques with large samples is attractive, and many labs worldwide use them for their studies in many different species^18–23^. Understanding how developmental processes take place in 3D will certainly extend our comprehension of how the brain develops in both healthy and diseased states.

## Deconstructing development one cell at a time

The complexity and diversity of cell types is one of the most remarkable characteristics of the mature nervous system. Corticospinal neurons that connect the brain with the spinal cord, sensory neurons that detect and conduct touch information from the skin surrounding our bodies to the central nervous system, or glial cells that modulate neural activity are different examples of the richness and huge variability in cell types that make up the nervous system. Cell specification occurs during development and, until recently, researchers had very limited ways to quantify cell diversity. For many years, the quantification of diversity was constrained by the use of pooling approaches and techniques that require either harvesting cells from the same tissue or combining cells from different individuals to obtain enough material for downstream analysis. For example, in bulk RNA sequencing (RNAseq) approaches, the transcriptomic expression level of a particular gene is not measured from an individual cell but rather as the average level of expression of that gene over many cells present within the same sample. The revolution in the molecular analysis of individual neural progenitors started when the ability to sequence DNA or RNA at the single-cell level became possible. In 2013, the journal *Nature Methods* highlighted the ability to sequence DNA and RNA in single cells as the “Method of the Year”^25^, and since then single cell approaches have been continuously developed to measure and characterise different aspects of cell identity (chromatin accessibility, the genome, transcriptome, and proteome). In fact, combinations of transcriptomics plus epigenomics or transcriptomics plus proteomics in single cell analyses are rapidly emerging^26,27^. Here, we focus our attention on one of the most frequently used modalities in neural development, the transcriptomic characterisation of single cells.

The first protocol to perform single cell RNAseq (scRNAseq) was published in 2009^28^, and the myriad of protocols that have been developed since then have quickly transformed several research fields and the way developmental studies are performed. The key step in scRNAseq protocols consists of tagging all transcripts inside each cell in such a way that RNA molecules coming from the same cell are easily identifiable and quantifiable^29^. scRNAseq enables transcriptomic cell types in the sampled tissue to be defined through the analysis of differentially expressed genes in each cell.

Nowadays, commercialisation of droplet-based sequencing, for example the 10x Genomic Chromium platform, has enabled the widespread use of scRNAseq. In the field of developmental neuroscience, scRNAseq has been used to profile the entire developing mouse brain and spinal cord^30^ as well as the prefrontal cortex of human embryos^31^ or the temporal changes in the transcriptional landscape of apical progenitors and their successive cohorts of daughter neurons in the cortex^32^. In general, developing tissues are characterised by the presence of a mix of cells in different stages of differentiation (progenitors, neuroblasts, early postmitotic neurons, and mature neurons). These stages are captured at the time of scRNAseq processing, thus resulting in a continuous representation of cellular states transitioning from one to another. These transitional stages may be modelled computationally by recapitulating the probable trajectory of the cells through a representation called pseudotime^33^, which defines the order of the cells through development. This representation therefore enables mapping of particular cell types to different states of the developmental trajectory^34^.

**Figure 2.**
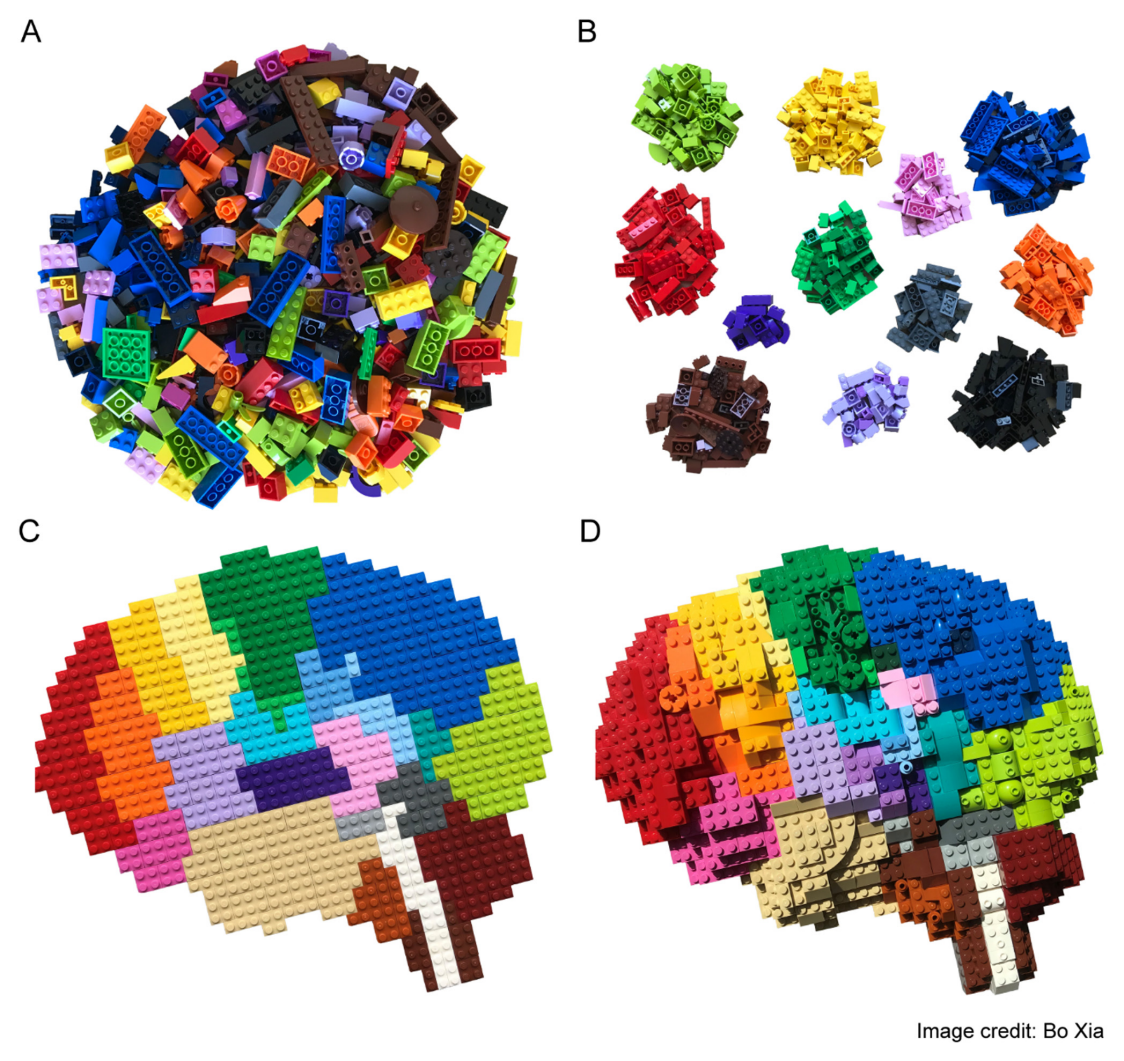
Building-block representation of single cell transcriptome modalities. **A.** Bulk RNA sequencing (RNAseq) experiments use a large number of cells as starting material, which results in a high depth and resolution at the transcriptomic level. However, because the measurements obtained represent an average of gene expression across all of the cells present in the sample, any differences between cells become occluded. **B.** Single cell RNAseq (scRNAseq) methods are capable of maintaining cell individuality during isolation of mRNA molecules. mRNAs are tagged and reconstructed informatically so that they can be assigned to a particular cell. This enables the identification of cell clusters according to their transcriptomic signatures, but spatial information is still lost. **C.** The spatial location of each cell is maintained in spatial transcriptomics approaches. By fluorescently tagging each mRNA species or recording the position *in situ* with barcodes, spatial information may be assigned to each cell together with their transcriptomic profile. Currently, the best attainable resolution is within the tens of microns range, which is still far from ideal and sensitivity remains low. As this is a novel method, the availability of the protocol is scarce and its adoption outside originator labs is therefore difficult. **D.** Researchers are now advancing towards an integrated (genetic, transcriptomic, and proteomic) representation of the brain in time and space. This figure has been reused with permission from the creator Bo Xia.

Unfortunately, this now-widespread technique has an important weakness: the loss of spatial information. Tissues, especially during development, are highly structured and dynamic, a fact that underpins the biological relevance of spatial information. The preparation of single cell suspensions needed to perform scRNAseq analyses requires the homogenisation of tissues and, as such, results in a loss of such spatial information. To overcome this limitation, several labs have now developed a series of methods commonly referred to as spatial transcriptomics. These methods vary in the way in which they maintain spatial information in the tissue sample as well as in their sensitivity, the number of transcripts that can be probed, and the spatial resolution attainable^35–39^, with the latest iteration of the high-definition spatial transcriptomics (HDST) method^40^ reaching a spatial resolution of 2 µm (Figure 2).

It is also worth mentioning that scRNAseq approaches can be similarly applied to single nuclei. The advantages become obvious when we consider that nuclei extraction is a routine method performed in molecular biology labs, that nucleic acids are stable in fixed and frozen samples, and that clinical human tissue banks from where nuclei can be easily obtained are abundant. Moreover, enzymatic dissociation during sample preparation is not required for single nuclei RNAseq (snRNAseq), thereby yielding cell types that are more representative of the original tissue and less affected by transcriptional artefacts. A potential disadvantage of using nuclei is that lower quantities of messenger RNA are obtained from the nuclei than from whole cells, and consequently fewer genes are typically detected. Nonetheless, recent studies have shown that single nuclei and single cell approaches identify similar cell types^41,42^.

## Exploiting artificial intelligence to understand neural development

The heading of this section might sound as if it were taken from a sci-fi movie. However, combining the computational power of modern processors and graphics processing units (GPUs) is exactly what high-throughput methodologies such as those described above require. These techniques generate huge quantities of data that need to be analysed. The simple generation of sequencing results or imaging data by itself does not provide new insights that can advance our understanding of how the nervous system develops. Intense developments in the field of machine learning have generated algorithms that may now be used to deconstruct the complexity of such data.

The imaging of a mouse brain by high-resolution LSFM generates between 20 gigabytes and up to several terabytes of data depending on the resolution^9^. Navigating your way through such an enormous amount of information to draw conclusions quickly becomes a dead-end, both in time and in computing requirements. Working with such vast quantities of bytes imposes a heavy processing burden on a lab’s computing capability but also makes analysing such datasets a time-consuming task. To solve this problem, the development of software capable of handling huge quantities of data becomes an urgent requirement; it becomes just as important as the need for the hardware that generates the dataset itself.

Paradoxically, even though a researcher spends just a few minutes to image a whole mouse embryo in 3D using LSFM, the quantification of such datasets often relies on tools and systems that require manual and time-consuming annotations. Fortunately, in the last few years, an unparalleled development of informatics tools has begun to help researchers quickly and accurately analyse big datasets in a short period of time. Some examples of these programs are *Cell Profiler* from the Broad Institute, which can easily segment nuclei in dense tissue images, and its more powerful sibling, *Cell Profiler Analyst*, which makes use of machine learning algorithms to recognise defined cell types from large imaging datasets^43,44^. *Cell Profiler* has been used, for example, to quantify the differences in neuronal numbers between the sulci and gyri of the cortex of Flrt3 mutant mouse embryos^45^ and to help elucidate the role of PTPRD in neurogenesis^46^. More recently, the Pachitariu lab^47^ released a complementary approach for cell segmentation called *Cellpose*. This is a generalist algorithm for cellular segmentation and is based on the use of a neural network that is trained on thousands of images from different microscope modalities (fluorescent, bright field, etc.) combined with non-biological images of similar structure. The system creates a platform capable of recognising cells from a wide array of image types. It also enables the generation of researcher-defined custom models by training the algorithm on specific types of images.

The use of machine learning, particularly neural networks trained to recognise structures of interest such as nuclei, cells, blood vessels, noise, etc. in images, has exploded in the last few years, and it is quickly becoming the go-to solution for many biomedical research problems^48–57^. Beyond solving imaging tasks, machine learning approaches may be used in many other applications within the field of neurodevelopment. The quantity of data generated during sequencing experiments such as scRNAseq face the same challenges as those derived from large imaging experiments. Newer and more refined technologies yielding an ever-increasing number of sequenced cells quickly translate not only into larger datasets but also into a higher number of dimensions that need to be non-linearly reduced to define particular cell types. Several packages that allow the processing of sequencing data and perform efficient dimensionality reduction or help to identify defined cell types of interest within the datasets have been released^58,59^. While processing of image and sequencing data are both examples from the blooming field of computational biology that are useful for studying neural development, many more developments and applications are predicted to emerge^60^.

## What lies ahead?

Although transformational technological revolutions are constantly occurring in science, the advances that have been made in the last few years have been spectacular. Here we have highlighted what, in our opinion, are very relevant and novel approaches for investigating the developmental processes that control the formation of neural circuits. It is our belief that we will experience amazing changes in the years to come that will dwarf what we know today. We envision that tissue clearing technologies will evolve into fully applicable methods that will no longer be limited by antibody compatibility. The recent publication of CUBIC-HistoVision points in that direction, as it describes a systematic interrogation of the properties and conditions that preserve antigens and facilitate antibody penetration into fixed animal tissues^61^. Community crowd-sharing of resources such as those mentioned, antibody-related optimisations, and tested protocol modifications and reagents will form the basis for advancing current and future protocols, likely to the point that many antibodies will work for 3D immunostaining applications. Concurrently with advancements in staining methods, parallel development of LSFM will likely enhance the imaging resolution of transparent samples while also reducing the time required for acquisition^62,63^.

The most important missing piece for single cell approaches is the development of high-throughput proteomics to individually measure protein content in each cell with enough depth to cover the whole proteome. Beyond basic estimation per cell, single cell DNA or RNA technologies are incapable of measuring the abundance and activity of proteins, which are regulated by both post-translational modifications and degradation. Although single cell proteomic approaches are already available, most of them currently rely on antibodies to detect the proteins of interest; this imposes an important throughput limitation. Methods to quantify thousands of proteins in hundreds of cells through the use of mass spectrometry (MS) are emerging, and improvements in MS are expected to increase the sensitivity of single cell proteomics^64^. The development of effective and high-throughput approaches in single cell proteomics will aid the quest to fully characterize cells, their functional and developmental states, and the mechanisms involved in transitioning from one state to another. Matched single cell genetic, transcriptomic, and proteomic data will help to elucidate the mechanisms behind the formation of a fully developed nervous system.

The application of machine learning in the field of developmental neuroscience is still in its infancy but will likely explode in the near future. Examples stemming from cancer research^65^, such as those using neural networks trained to identify different types of tumours based on their location and composition in cleared whole mouse bodies^66^, highlight the possibilities of gathering current computing power so that it can be applied to other fields such as neural development. Similar applications of machine learning algorithms could aid in the recognition of changing mRNA/protein expression patterns in brain development. Labour-intensive tasks commonly used to study the developing nervous system could also greatly benefit from the implementation of tools developed in other neuroscience-related areas. For example, the automated identification and tracking of migrating neurons should be easily adopted following the lead of algorithms like DeepLabCut that behavioural neuroscience labs are using to track the position of different parts of the mouse body without the use of markers^67^. ClearMap is another algorithm that maps cells automatically in the mouse brain of LSFM datasets, which could be applied to neonate brains^24^. Another very promising avenue is the algorithm Trailmap, which was recently developed in the Luo lab to automatically identify and extract axonal projections in 3D image volumes^68^ and may be easily implemented to improve the quantification of axon guidance studies. Adoption of such neural networks will probably require re-training and optimisation to the specific use-case scenario and dataset, which highlights the need for fast-training computational strategies in order to facilitate the broader use of these techniques.

Therefore, despite the impressive amount of state-of-the-art technologies developed in the last few years, there is still room for improvement of some of the latest methods available to study the developing nervous system. We could envision a not-so-distant day when 3D embryonic brain imaging will be combined with single-cell technologies to elucidate the chromatin, mRNA, and protein signatures of each cell *in situ* at the same time*.* Such datasets would contain information about what are considered the main determinants of cell identity while maintaining the intact structure, shape, and form of the tissue. This “fantasy technical improvement” could be pictured even one step further by introducing the fourth dimension into account and analyse datasets of embryos at different stages of development to provide the most detailed description of development progression to date. However, writing a “what will the future look like” piece is bound to fail. History has demonstrated that both the imagination and the driving force of scientists are many orders of magnitude beyond what can be anticipated. As such, while this review will likely become obsolete shortly, it will be a good sign that developmental neuroscience maintains its exponential progression in advancing our understanding of the assembly of neural circuits.
